# Cyclin Dependent Kinase Inhibitor 2A Genetic and Epigenetic Alterations Interfere with Several Immune Components and Predict Poor Clinical Outcome

**DOI:** 10.3390/biomedicines11082254

**Published:** 2023-08-11

**Authors:** Mohamed A. Soltan, Ahmad A. Alhanshani, Ayed A. Shati, Youssef A. Alqahtani, Dalal Sulaiman Alshaya, Jawaher Alharthi, Sarah Awwadh Altalhi, Eman Fayad, Mohamed Samir A. Zaki, Refaat A. Eid

**Affiliations:** 1 Department of Microbiology and Immunology, Faculty of Pharmacy, Sinai University, Ismailia 41611, Egypt; 2 Department of Child Health, College of Medicine, King Khalid University, Abha 62529, Saudi Arabia; 3 Department of Biology, College of Science, Princess Nourah bint Abdulrahman University, P.O. Box 84428, Riyadh 11671, Saudi Arabia; 4 Department of Biotechnology, College of Sciences, Taif University, P.O. Box 11099, Taif 21944, Saudi Arabia; 5 Anatomy Department, College of Medicine, King Khalid University, Abha 62529, Saudi Arabia; 6 Pathology Department, College of Medicine, King Khalid University, Abha 62529, Saudi Arabia

**Keywords:** CDKN2A, differential expression, genetic alteration, epigenetic alteration, patient survival, immunotherapy

## Abstract

Cyclin dependent kinase inhibitor 2A (CDKN2A) is a well-known tumor suppressor gene as it functions as a cell cycle regulator. While several reports correlate the malfunction of CDKN2A with the initiation and progression of several types of human tumors, there is a lack of a comprehensive study that analyzes the potential effect of CDKN2A genetic alterations on the human immune components and the consequences of that effect on tumor progression and patient survival in a pan-cancer model. The first stage of the current study was the analysis of CDKN2A differential expression in tumor tissues and the corresponding normal ones and correlating that with tumor stage, grade, metastasis, and clinical outcome. Next, a detailed profile of CDKN2A genetic alteration under tumor conditions was described and assessed for its effect on the status of different human immune components. CDKN2A was found to be upregulated in cancerous tissues versus normal ones and that predicted the progression of tumor stage, grade, and metastasis in addition to poor prognosis under different forms of tumors. Additionally, CDKN2A experienced different forms of genetic alteration under tumor conditions, a characteristic that influenced the infiltration and the status of CD8, the chemokine CCL4, and the chemokine receptor CCR6. Collectively, the current study demonstrates the potential employment of CDKN2A genetic alteration as a prognostic and immunological biomarker under several types of human cancers.

## 1. Introduction

The condition of tumorigenesis is a complex one where a malfunction of specific genes, that regulate normal cell proliferation, allows for uncontrolled proliferation that finally might progress into tumor [1,2]. Under normal conditions, a group of genes named tumor suppressor genes, keeps the normal cell cycle and when these genes are subject to deleterious gene alteration, abnormal cell proliferation occurs as an early stage of carcinogenesis [3]. While specific gene upregulation has been correlated with a specific type of human tumor, several studies have been performed to dig for the genes that can be correlated with several forms of human tumors to generate general markers and therapeutic targets in a pan-cancer model [4]. The continuous development of cancer-related databases and bioinformatics tools contributed largely to the screening of tumor markers and therapeutic targets in a time-saving manner [5,6]. The currently available databases and tools provide valuable data regarding differential gene expression under tumor and normal status, the correlation of these genes with tumor stage, grade, metastasis, and the clinical outcome [7]. In addition to that, the effect of genetic alterations, which may occur to a specific gene, on cancer stimulation and progression become also available for deep analysis [8]. In the last few years, the field of tumor immunotherapy has witnessed a great revolution in the development of immune checkpoint inhibitors (ICIs), such as α PD-1 and α CTLA-4, that showed promising results with clinical application [9]. Consequently, the interference of cancer-regulating genes with the human immune components became a major point of research in cancer studies [10].

Tumor suppressor genes have vital roles in regulating cell division and maintaining genome integrity [11]. A certain balance between tumor suppressor genes and proto-oncogenes is always kept under normal cell conditions while the occurrence of progressive genetic and epigenetic alterations in those genes transforms the cell into a cancerous condition [12]. Genetic mutations and gene copy number variation (CNV) are categorized as major forms of genetic alteration that usually happen to tumor suppressor genes for the onset and progression of carcinogenesis while DNA methylation and histone acetylation are the main categories of the epigenetic changes [13,14]. As a result of these alterations, the functions of tumor suppressor genes in apoptosis induction, cell cycle regulation, and metastasis suppression are adversely affected [15] and that was reflected in carcinogenic manifestations, such as the increased risk of relapse and death in prostate cancer [16], poor clinical outcome in malignant pleural mesothelioma [17], and stimulated metastasis with impaired mitophagy in breast cancer [18]. Due to their important roles in the cancer fields, alterations in tumor suppressor genes have been detected as a major target for drug development and while numerous attempts experienced failure in the past, continuous research is running to investigate a novel approach that could restore the level and function of the tumor suppressor genes as advances in cancer genomics can finally provide an effective treatment targeting these genes [19].

The CDKN2A gene is a well-defined tumor suppressor gene located on chromosome 9p21 and responsible for the generation of two tumor suppressor proteins namely p16 and p14 [20]. P16 demonstrates its tumor suppressive activity through the inhibition of cyclin-dependent kinase CDK4 and CDK6 expression and limiting retinoblastoma protein phosphorylation during the G1/S cell cycle checkpoint [21]. For further confirmation, tumors with reported p16 deletion showed an enhanced ability to overcome cellular senescence and apoptosis in comparison to the tumors with wild-type p16 [22]. Therefore it is not a surprise that CDKN2A was reported to be the most common inactivated tumor suppressor gene in melanoma [23,24]. Deletion mutation of CDKN2A was reported in 31 cases with lung cancer where these cases also experienced EGFR mutations and the alteration in these genes was correlated with a poor response to anticancer treatment [25]. Moreover, the status of CDKN2A deletion was reported in lymphoma patients where cases with CDKN2A and TP53 dual deletion have experienced a lower survival in comparison to cases with a single gene deletion [26]. It is worth mentioning that the condition of CDKN2A deletion has been reported in other types of human cancers including glioblastoma [27], sarcoma [28], and gastric carcinoma [29].

While the genetic alterations in CDKN2A have been reported in several single forms of human cancers, there is a lack of a comprehensive study that deeply describes the state and the type of the genetic and epigenetic modifications of CDKN2A in a panel of human tumors. Additionally, the correlation between CDKN2A and the components of the immune system under tumor conditions is still not fully described. Understanding these factors could direct cancer research in biomarkers discovery and therapeutic target identification. The current study sought to investigate the expression status of CDKN2A in a panel of human tumors and study the possible correlation with tumor grade, stage, metastasis, and clinical outcome. The study also aimed to identify the DNA methylation status of CDKN2A, as an important form of epigenetic regulation, investigate the shapes of CDKN2A genetic alterations, and study the outcome of these alterations on the human immune cells and cytokines that play major roles in tumor progression.

## 2. Materials and Methods

### 2.1. CDKN2A Differential Expression Analysis

The current study started by analyzing the differential expression of CDKN2A for tumor tissues and normal ones where the data deposited in the TIMER2 web server [30] was used for that purpose through the accession of the “GENE_DE” model under the “Cancer Exploration” panel. During the current analysis and relying on the data from TIMER2, several tumors experienced a lack of normal tissue for the comparative analysis of CDKN2A expression, therefore, another database, namely GEPIA2 [31], was employed to complete the analysis where we used “Match TCGA normal and GTEx data” as the selected matched normal data and 0.01 as the *p*-value cut-off. Following the differential expression assessment, we asked about the effect of differential CDKN2A levels under the tumor conditions on tumor stage, grade, and metastasis, and for that purpose, we used TISIDB [32] and TNMplot [33] repositories under “clinical” and “gene expression comparison” panels, respectively.

### 2.2. Assessment of Differential CDKN2A Protein Levels

In order to investigate the differential CDKN2A protein levels in a panel of human tumors, the UALCAN tool was utilized [34] using data from Clinical Proteomic Tumor Analysis Consortium (CPTAC). Moreover, the human protein atlas (HPA) provides IHC images for several proteins under tumor and normal conditions [35], therefore this database was used to obtain the staining images of the protein of interest, through the accession of “tissue” and “pathology” tabs, to confirm the generated data from the UALCAN tool.

### 2.3. Survival Analysis

In order to study the correlation between CDKN2A and the clinical outcome we used two databases, namely GEPIA2 [31] and KM plotter [36]. Under GEPIA2, CDKN2A was assessed for the correlation between its level and two survival modes namely overall survival and disease-free survival, through the accession of the “survival analysis” tab. Regarding the KM plotter, pan-cancer RNA seq was selected to investigate the correlation between CDKN2A levels and the overall survival for 7462 cases and relapse-free survival for 4420 cases.

### 2.4. Investigation of CDKN2A Genetic Alterations under Tumor Conditions

At this stage, we accessed the cBioPortal database [8] to perform a deep analysis of the reported CDKN2A genetic alterations. The analysis started with the investigation of the forms of CDKN2A genetic alterations in a list of human cancers under the “Cancer Types Summary” panel. Following that, the types and sites of CDKN2A-reported mutations were analyzed under the “Mutations” panel. Finally, we studied the effect of these alterations on the clinical outcome by investigating the data under the “Comparison/Survival” panel.

### 2.5. Epigenetic Modulation of CDKN2A under Tumor Conditions

It has been reported that tumor suppressor genes are subjected to an epigenetic modulation to control their expression under tumor conditions where DNA methylation represented a major approach for that modulation [37]. Consequently, we employed two portals namely UALCAN [38] and SMART app [39] under “TCGA Gene” and “CpG-aggregated methylation”, respectively, to investigate the DNA methylation status of CDKN2A under tumor conditions and compare that with normal controls.

### 2.6. Interference of Altered CDKN2A with the Infiltration and Status of Different Immune Components

The role of the human immune system has been deeply studied for its effect on tumor growth where several cells and chemokines with opposed effects have been identified [40,41]. For that purpose, we investigated the effect of CDKN2A genetic alteration under tumor conditions on several immune components. Because CD8 T cell has a major role in fighting against cancer, it was the first immune component to be analyzed. Under chronic stimulation, CD8 T cells were reported to obtain a dysfunctional status of exhaustion which is marked by the expression of several immune checkpoint inhibitors [42], hence not only the infiltration but the status of the CD8 T cell was studied for the possible correlation with CDKN2A alteration. TIMER2 web server [30] was employed to find the correlation between CDKN2A alteration and the infiltration of CD8 T cells through the accession of the “sCNA” panel under “Immune Association” and selecting “Deep Deletion” as the alteration status for comparison. Additionally, the TISIDB repository was utilized to investigate the correlation between CDKN2A alteration with the expression of several immune checkpoint inhibitors (under the immunomodulator tab) in addition to the expression of the chemokine CCL4 and the chemokine receptor CCR6 (under chemokine tab) which are known for immunosuppressive roles allowing for cancer progression [43,44].

### 2.7. CDKN2A Enrichment Analysis

Here we aimed to investigate the interaction network of CDKN2A to predict how the genetic alteration of the CDKN2A could affect that network and consequently the cell condition. Firstly, a list of CDKN2A interacting proteins was obtained using the STRING database [45]. We obtained the top 50 interacting proteins by setting “Experiments” as the active interaction source and “low confidence” as the interaction score. Following that, the GEPIA2 repository [31] was employed to obtain the top 100 correlated genes to CDKN2A under tumor conditions based on the analysis of TCGA tumors where a Venn diagram was generated through the web server (http://bioinformatics.psb.ugent.be/webtools/Venn/, accessed on 1 March 2023) was used to visualize the common genes in the above two generated lists. Finally, a common list of the combined interacting and correlated genes was submitted to the DAVID database [46] to run an enrichment analysis and the results were visualized using the “ggplot2” package of R (4.2.0).

## 3. Results

### 3.1. CDKN2A Upregulation in Tumor Tissue Is Positively Correlated with the Stage, Grade, and Metastasis of Specific Human Tumors

**Figure 1:**
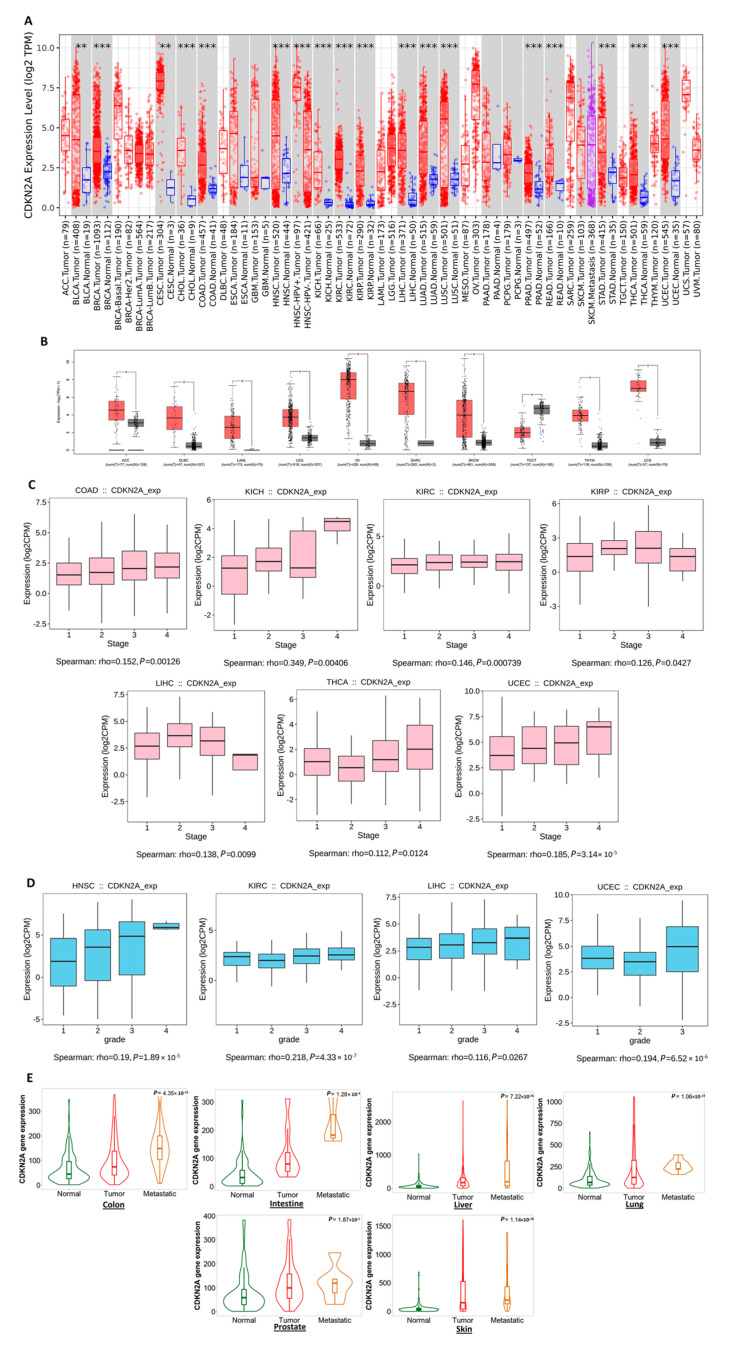
CDKN2A differential expression analysis and its interference with tumor stage, grade, and metastasis. (**A**) Differential expression of CDKN2A as analyzed by TIMER2.0 where blue represents normal, red represents tumor, and purple represents metastatic sample (**: *p*-value < 0.01; ***: *p*-value < 0.001) (**B**) GEPIA2 differential expression analysis of CDKN2A in tumors that lack a normal control on TIMER2.0 (*: *p*-value < 0.05). (**C**) Tumors experienced a positive correlation between CDKN2A levels and tumor stage. (**D**) Tumors experienced a positive correlation between CDKN2A levels and tumor grade. (**E**) Tumors experienced a consistent positive correlation between CDKN2A expression and tissue type (normal–tumor–metastatic).

The differential expression of CDKN2A was firstly assessed using the TIMER2 repository and the output revealed that except for PAAD and PCPG, all of the analyzed tumors with normal tissue for comparison exhibited a significant upregulation of CDKN2A in tumor tissue versus control (Figure 1A). It is worth mentioning that the number of normal samples for comparison in PAAD and PCPG was four and three, respectively, and this low number could explain why the significant correlation was absent. Next, we employed the GEPIA2 database to investigate the differential expression of CDKN2A in the tumors without a control in TIMER2 and the results revealed that nine tumors, namely ACC, DLBC, LAML, LGG, OV, SARC, SKCM, THYM, and UCS, have experienced a significant CDKN2A upregulation in cancerous tissues versus normal ones (Figure 1B). After confirming the significant upregulation of CDKN2A in most of the analyzed tumors, we asked if that upregulation could affect tumor progression. The output from TISIDB and TNMplot repositories revealed that seven tumors, namely COAD, KICH, KIRC, KIRP, LIHC, THCA, and UCEC, have demonstrated a significant positive correlation between CDKN2A levels and the tumor stage (Figure 1C), while HNSC, KIRC, LIHC, and UCEC showed a significant positive correlation between CDKN2A expression and the tumor grade (Figure 1D). The final assessment at this stage was the correlation between CDKN2A and tumor metastasis where six human tissues (colon, intestine, liver, lung, prostate, and skin) demonstrated a significant upregulation in CDKN2A levels in cancerous tissue versus normal one and this upregulation was consistent in metastatic tissue in comparison to normal and cancerous ones (Figure 1E).

### 3.2. CDKN2A Differential Protein Level Analysis and IHC Staining

**Figure 2:**
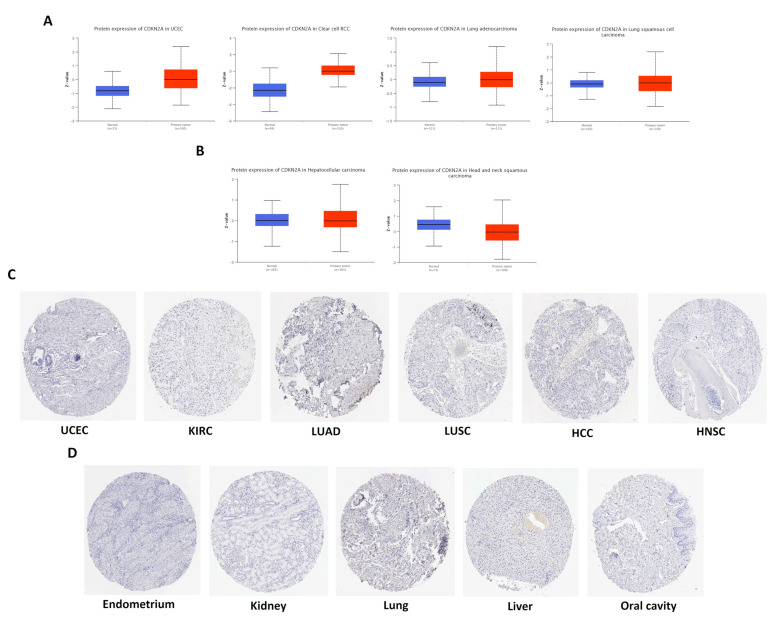
(**A**) Tumors experienced significant upregulation of CDKN2A protein in tumor tissue versus control. (**B**) Tumors experienced significant downregulation of CDKN2A protein in tumor tissue versus control. (**C**,**D**) IHC samples showing weak or no stain for CDKN2A protein in tumor and normal samples, respectively where all samples are downloaded with 200 µm scale.

After exploring the differential transcriptional levels of *CDKN2A* in cancerous and normal tissue and observing the upregulation of CDKN2A under tumor conditions, we aimed to study the protein levels to investigate if a similar pattern will be found. An analysis of CDKN2A protein levels based on the CPTAC dataset revealed that only four tumors (UCEC, KIRC, LUAD, and LUSC) have experienced a significant upregulation in CDKN2A protein levels in comparison to the corresponding normal samples (Figure 2A). On the other hand, HCC and HNSC showed significant downregulation of CDKN2A in comparison to the corresponding controls (Figure 2B). To confirm these findings, the HPA was employed to investigate the staining of CDKN2A protein in the tumors that experienced a significant upregulation or significant downregulation of CDKN2A protein levels, and the staining of the same protein in the corresponding normal tissue was also obtained for comparison. By tracking the staining with antibody CAB000093, approximately half of the deposited staining samples of UCEC showed low or not detected staining of CDKN2A. Moreover, CDKN2A staining was not detected in approximately 70% of LUAD, LUSC, and HNSC samples. CDKN2A staining was also not detected in 90% and 100% of HCC and KIRC samples. Collectively, CDKN2A staining was not detected in most of the analyzed tumor samples (Figure 2C), which resembled the staining status of the corresponding normal tissues (Figure 2D).

### 3.3. CDKN2A Overexpression Predicts a Poor Clinical Outcome in Several Human Tumors 

**Figure 3:**
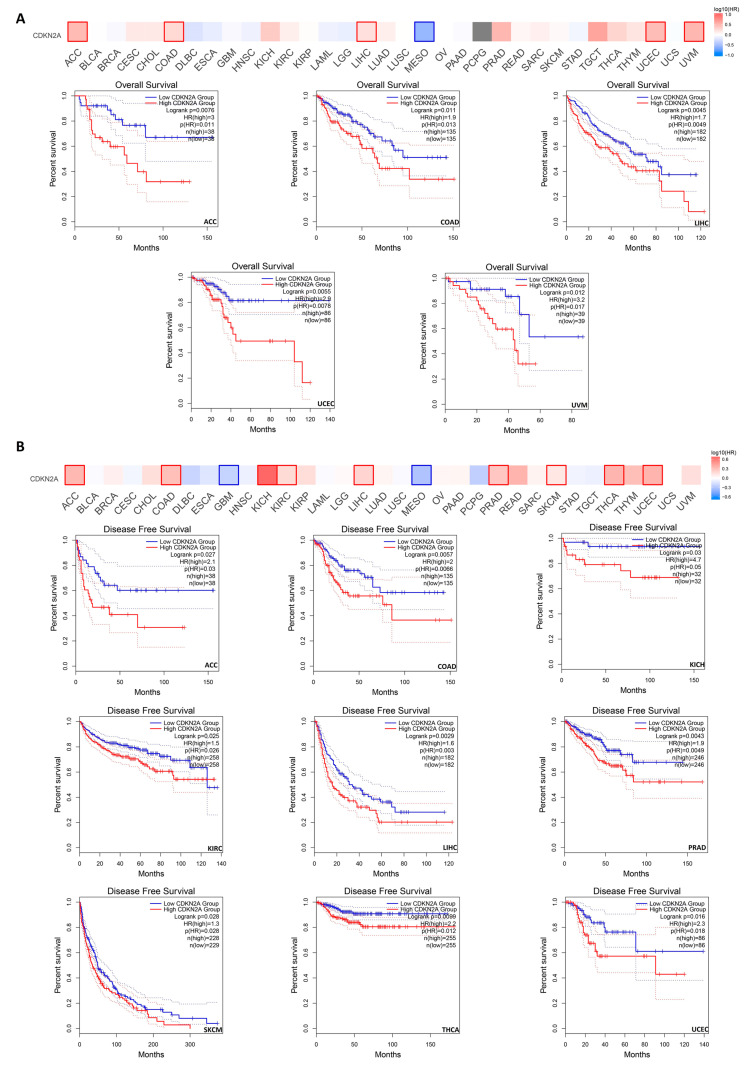
The correlation between CDKN2A expression and patient survival. (**A**) Disease-free survival. (**B**) Overall survival as assessed from the GEPIA2 database.

**Figure 4:**
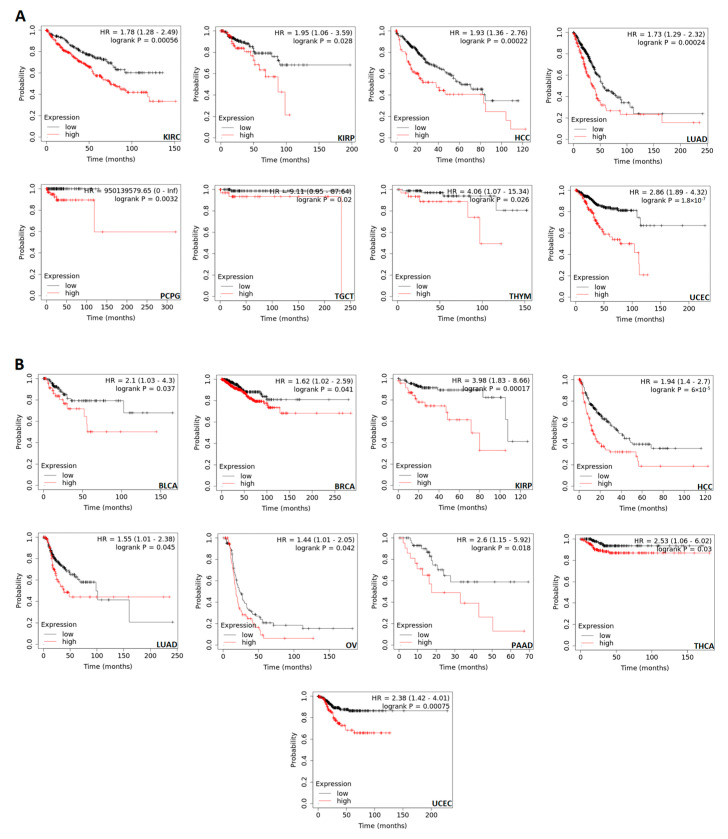
The correlation between CDKN2A expression and patient survival. (**A**) Overall survival. (**B**) Relapse-free survival as assessed from the KM plotter.

The correlation between CDKN2A overexpression and the clinical outcome was investigated through two databases. Analysis in the GEPIA2 database showed that five tumors namely ACC, COAD, LIHC, UCEC, and UVM have experienced a negative correlation between CDKN2A levels and overall survival (Figure 3A). Additionally, nine tumors namely ACC, COAD, KICH, KIRC, LIHC, PRAD, SKCM, THCA, and UCEC demonstrated a negative correlation between CDKN2A levels and disease-free survival (Figure 3B). Moving to the results of the KM plotter, the second employed database, a negative correlation between CDKN2A levels and overall survival was found in KIRC, KIRP, HCC, LUAD, PCPG, TGCT, THYM, and UCEC (Figure 4A). A similar finding was observed in BLCA, BRCA, KIRP, HCC, LUAD, OV, PAAD, THCA, and UCEC when relapse-free survival was assessed (Figure 4B). 

### 3.4. CDKN2A Genetic Alteration Adversely Affects Patient’s Survival

**Figure 5:**
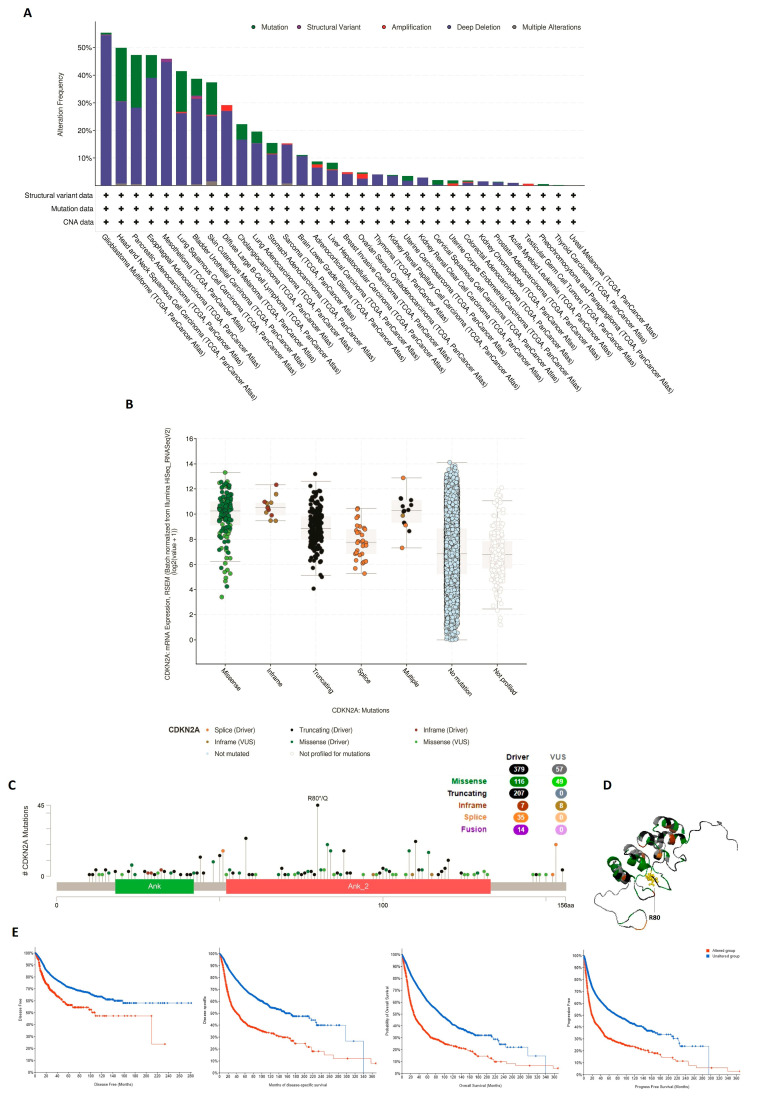
Mutation profile of CDKN2A as assessed by the cBioPortal web server. (**A**) The alteration frequency of CDKN2A in a panel of analyzed human cancers. (**C**) Forms of CDKN2A mutations. (**B**) A map for sites and types of CDKN2A mutations. (**D**) The 3D structure of CDKN2A, with a highlight on the most altered site. € Assessment of the correlation between CDKN2A mutation and disease-free, disease-specific, progression-free, and overall survival.

It is repeatedly reported that tumor suppressor genes suffer from genetic alterations under the tumor conditions, therefore the cBioPortal database was utilized to investigate CDKN2A genetic alterations. Most of the analyzed tumors experienced “deep deletion” and “mutation” as the first and second most common forms of CDKN2A genetic alterations (Figure 5A). GBM, HNSC, PAAD, and ESCA were the top human tumors to experience CDKN2A genetic alterations with a frequency of approximately 50%. Moreover, while some tumors, such as GBM, MESO, and SARC, have experienced “deep deletion” as approximately the only form of genetic alteration, others, including HNSC, PAAD, and LUSC, experienced “mutation” in addition to “deep alteration” as the major forms of CDKN2A genetic alterations (Figure 5A). Focusing on CDKN2A mutations, missense, and truncating mutations were the dominant forms (Figure 5B). Investigation of the mutation sites on CDKN2A demonstrated that the amino acid number 80 was the most common site of reported mutations (Figure 5C,D). Most importantly, these genetic alterations highly influenced the clinical outcome negatively where disease-free, disease-specific, progress-free, and overall survivals were negatively affected in altered groups in comparison to unaltered ones (Figure 5E).

### 3.5. CDKN2A Hypermethylation in Most of the Analyzed Human Tumors

**Figure 6:**
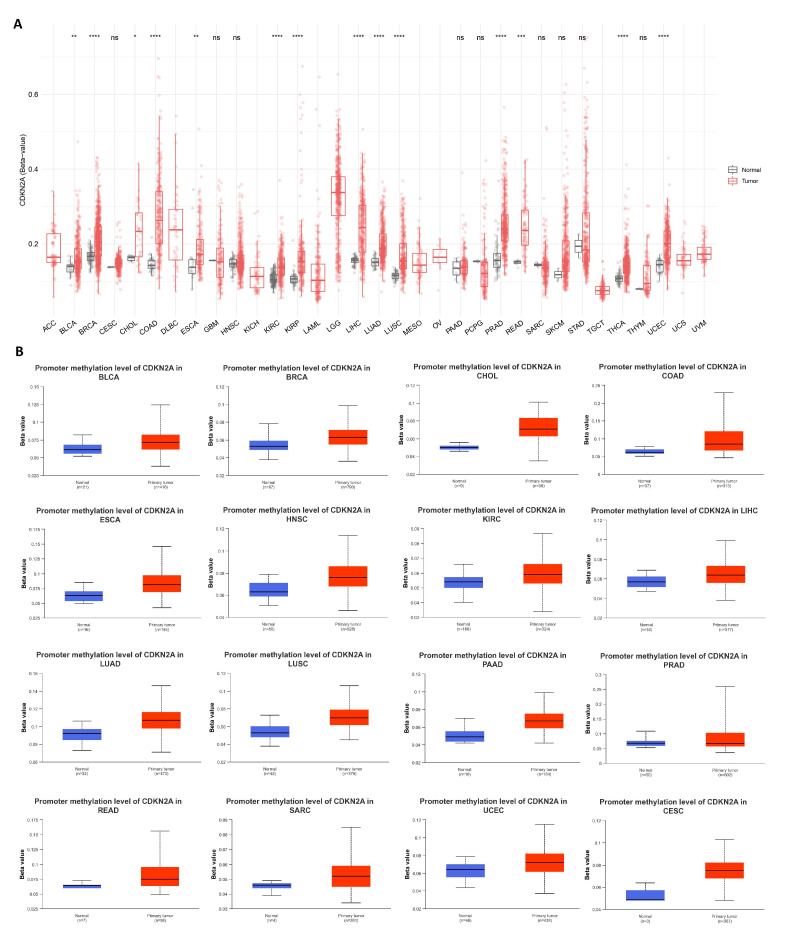
Differential methylation analysis of CDKN2A in tumor samples versus normal ones. (**A**) Analysis of CpG-aggregated methylation of CDKN2A. ns: *p* > 0.05; *: *p* ≤ 0.05; **: *p* ≤ 0.01; ***: *p* ≤ 0.001; ****: *p* ≤ 0.0001. (**B**) Tumors with CDKN2A promoter hypermethylation in tumor samples versus control.

After the assessment of CDKN2A genetic alterations, it was important to explore the epigenetic modifications that occur in CDKN2A to control its expression under tumor conditions. At this stage, we were concerned about DNA methylation as an important cellular mechanism for the epigenetic modification of a specific gene. The output of the CDKN2A CpG-aggregated methylation revealed that all of the analyzed tumors with significant results experienced hypermethylation in comparison to normal controls (Figure 6A). Moving to the promoter methylation level assessment, almost all of the analyzed tumors with a significant score (*p*-value < 0.05) demonstrated hypermethylation of CDKN2A under tumor conditions in comparison to normal samples (Figure 6B).

### 3.6. Genetic Alterations in CDKN2A Stimulate Immunosuppressive Components and Inhibit Cytotoxic CD8 T Cell Infiltration

**Figure 7:**
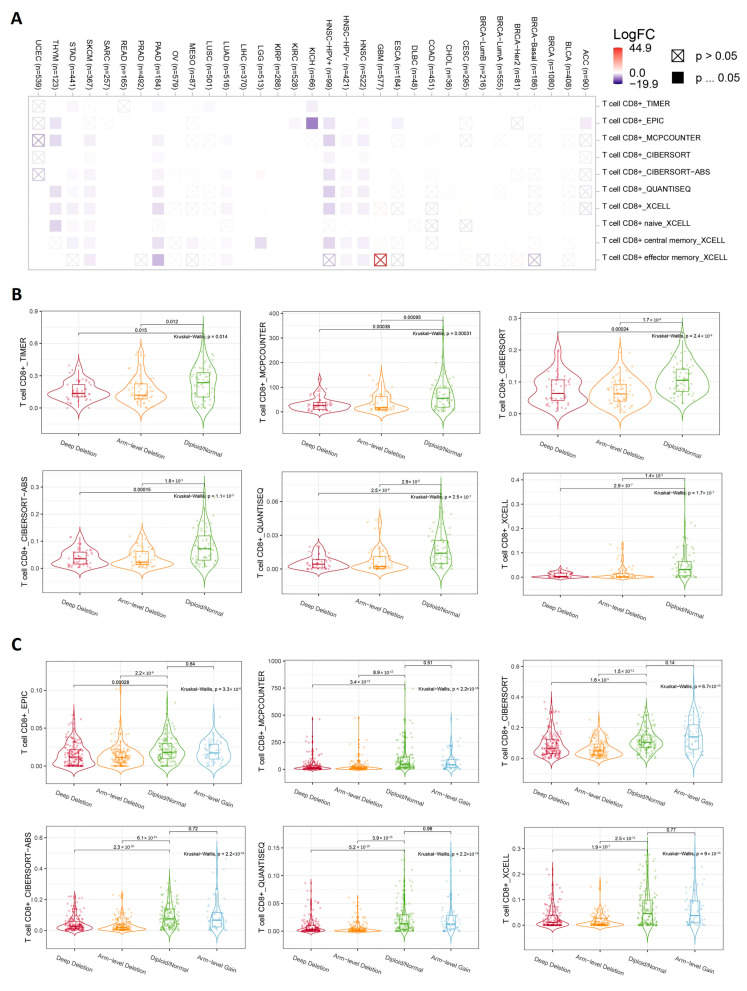
(**A**) Heat map showing the correlation between CDKN2A deep deletion and CD8 infiltration. (**B**,**C**) Violin plots showing the correlation between CDKN2A deletion and CD8 infiltration in PAAD and HNSC, respectively.

**Figure 8:**
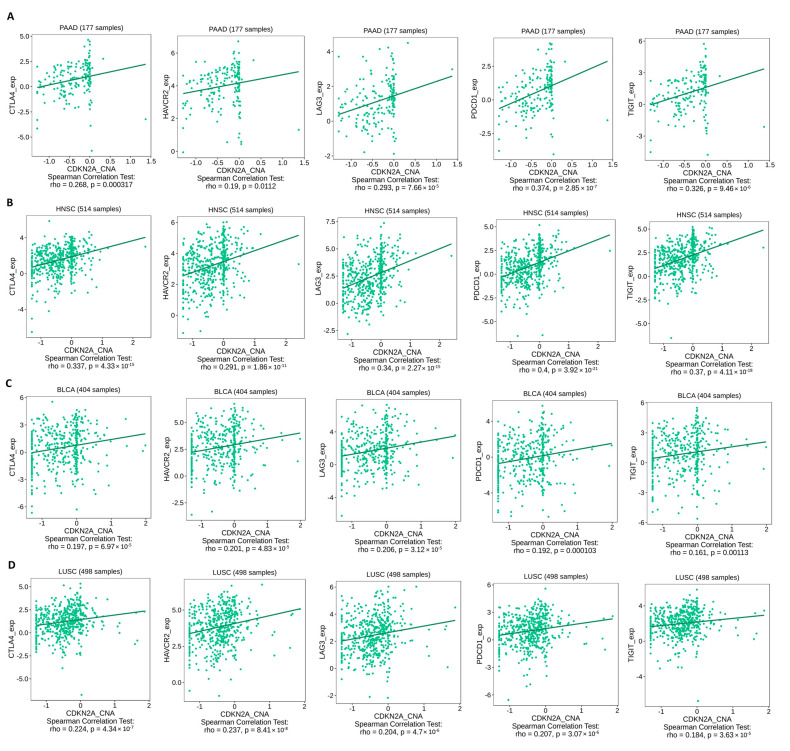
Scatter plot showing the positive correlation between CDKN2A CNA and the expression of several immune checkpoints in (**A**) PAAD, (**B**) HNSC, (**C**) BLCA, and (**D**) LUSC.

**Figure 9:**
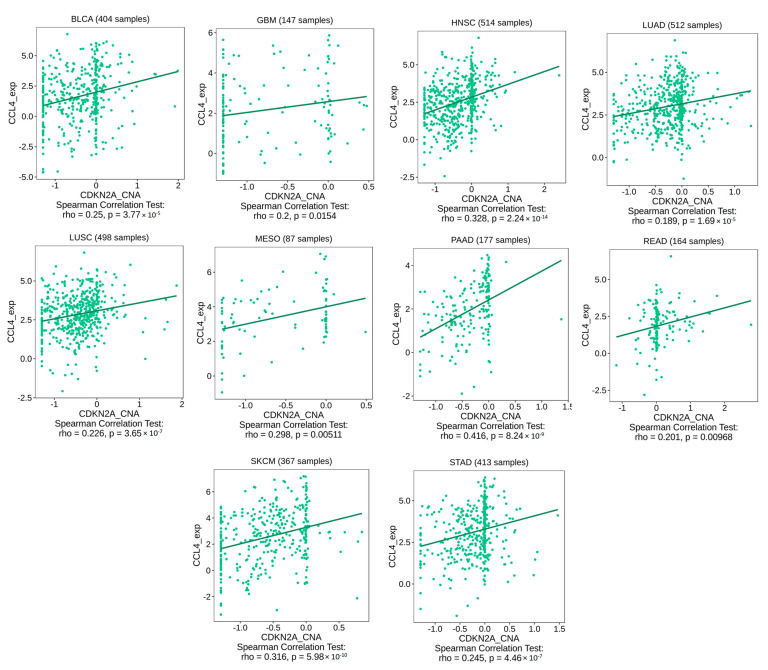
Tumors that experienced a positive correlation between CDKN2A CNA and CCL4 expression.

**Figure 10:**
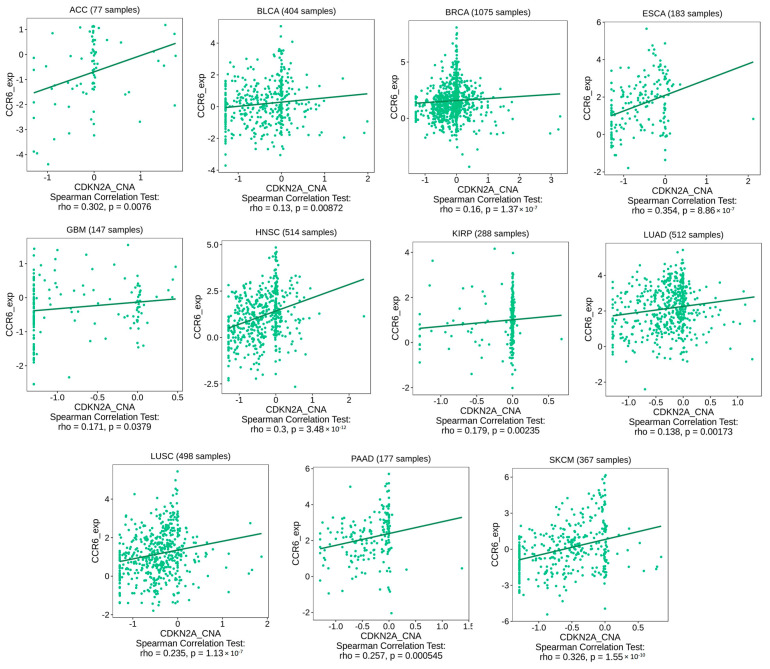
Tumors that experienced a positive correlation between CDKN2A CNA and CCR6 expression.

After confirming the state of the genetic alteration of CDKN2A and its negative impact on the patient’s survival, we asked if the immune components are correlated with that. Because “deep alteration” was the most common form of CDKN2A genetic alteration (Figure 5A), the current assessment started by studying the correlation between CDKN2A deep deletion and the infiltration of CD8 T cells where several tumors, including HNSC, KICH, LGG, PAAD, SKCM, STAD, and THYM, showed a negative correlation between CDKN2A deep deletion and the infiltration of different CD8 T cell subsets (Figure 7A). There were two tumors, namely PAAD (Figure 7B) and HNSC (Figure 7C), that showed this negative correlation in 6 out of 7 applied algorithms. After exploring the effect of CDKN2A alteration on the CD 8 T cell infiltration, we asked if that alteration could also affect the exhaustion status of the CD8 and, consequently, its effector functions. The expression of several immune checkpoints is a basic marker for CD8 exhaustion, therefore we explored the correlation between CDKN2A copy number alteration (CNA) and the expression of several immune checkpoints, including CTLA-4, TIM-3, LAG-3, PD-1, and TIGIT. We selected four tumors for that analysis and two of them (PAAD and HNSC) experienced a negative correlation between CDKN2A alteration and CD8 infiltration, while the other two tumors (BLCA and LUSC) did not experience that correlation. The output of this assessment revealed that all four analyzed tumors experienced a positive correlation between CDKN2A CNA and the expression of targeted immune checkpoints (Figure 8). Collectively, alteration of CDKN2A can affect both the infiltration and the status of the CD8 T cells. Next, we asked if CDKN2A alteration could affect other immune components; therefore, we checked the correlation of CDKN2A alteration with the chemokine CCL4 and the chemokine receptor CCR6, which are known for their immunosuppressive roles against tumor development. Our analysis revealed that 10 tumors, namely BLCA, GBM, HNSC, LUAD, LUSC, MESO, PAAD, READ, SKCM, and STAD, experienced a positive correlation between CDKN2A CNA and the expression of CCL4 (Figure 9). Furthermore, 11 tumors, namely ACC, BLCA, BRCA, ESCA, GBM, HNSC, KIRP, LUAD, LUSC, PAAD, and SKCM, exhibited a positive correlation between CDKN2A CNA and the expression of CCR6 (Figure 10).

### 3.7. CDKN2A Related Genes Enrichment Analysis

**Figure 11:**
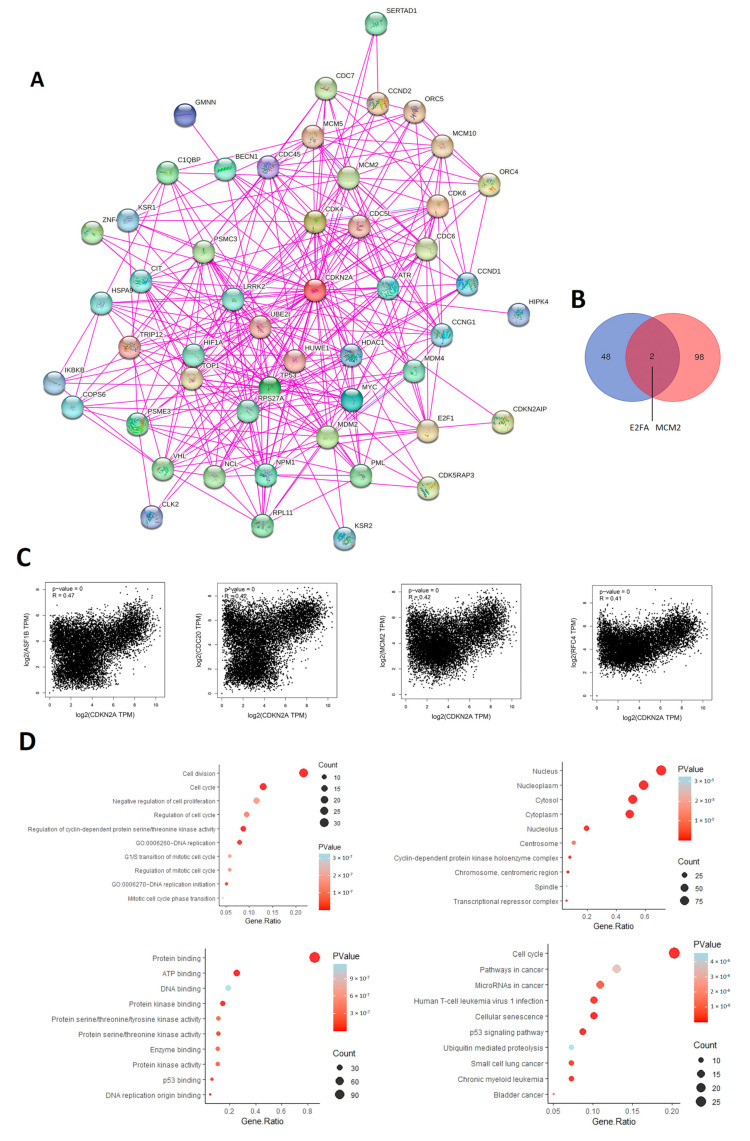
CDKN2A network interactions. (**A**) Network of top 50 CDKN2A interacting proteins as determined by the STRING database. (**B**) Venn diagram demonstrating the intersection between CDKN2A interacting and correlating proteins. (**C**) Scatter plot showing the positive correlation between CDKN2A expression and the expression of (ASF1B, CDC20, MCM2, and RFC4) as determined by GEPIA2. (**D**) KEGG/GO enrichment analysis based on CDKN2A-binding and interacted genes.

To investigate the consequences of CDN2A genetic alterations on its molecular circuit and the output cellular function, we performed an enrichment analysis for CDKN2A-related genes. Firstly, we obtained the top 50 interacted genes with CDKN2A from the STRING database (Figure 11A). Secondly, the GEPIA2 database provided the top 100 correlated genes with CDKN2A under tumor conditions where the correlation plots of the top four true genes are shown in Figure 11C. It is worth mentioning that two proteins, namely E2FA and MCM2, were detected as common genes in the generated lists of the two employed databases (Figure 11B). Lastly, a list of genes, that combines CDKN2A interacted and correlated genes, was submitted for enrichment analysis. These genes were enriched for “nucleus” and “nucleoplasm” regarding the cellular component and enriched for “cell division” regarding the biological process (Figure 11D). Importantly, the output of the KEGG enrichment analysis revealed that the selected genes were enriched for “cell cycle”, “pathways in cancer” and “miRNAs in cancer” while the molecular function enrichment presented “protein binding” as the top enriched one (Figure 11D). Hence the enrichment analysis confirms the interaction of CDKN2A with other proteins to regulate the cell cycle and the malfunction of CDKN2A through the genetic alteration would interfere with several molecular components moving the cell towards tumorigenesis.

## 4. Discussion

Carcinogenesis is a complex process that involves several components to drive the cell from its normal state into a cancerous one [47]. CDKN2A is a gene that belongs to the category of tumor suppressor genes and the malfunction of these genes participates in tumor initiation [2]. The state of CDKN2A genetic and epigenetic alterations has been reported in several forms of human tumors [48,49]; therefore it is important to define the different shapes of these alterations, investigate their consequences on tumor progression and patient survival, and study the modifications that occur to the immune system components as a result of those alterations. Because CDKN2A alterations have been reported in different types of human tumors, the current study was performed in a pan-cancer model.

The current study started with the differential expression analysis of CDKN2A in cancerous tissues and normal ones and a significant upregulation of CDKN2A transcripts was observed in different analyzed tumors. This CDKN2A transcript upregulation was positively correlated with the stage of COAD, KICH, KIRC, KIRP, LIHC, THCA, and UCEC. Additionally that upregulation also positively correlated with the grade of HNSC, KIRC, LIHC, and UCEC. While CDKN2A transcripts levels were upregulated in most of the analyzed tumors, the assessment of protein expression revealed that only four tumors, namely UCEC, KIRC, LUAD, and LUSC, experienced upregulation in CDKN2A protein levels in cancerous tissues versus control while two tumors, namely HCC and HNSC, experienced the opposite pattern. This finding was confirmed through the assessment of the IHC samples deposited in the HPA. Matching with the findings in CDKN2A protein expression levels assessment, most of the deposited samples for HCC and HNSC showed no detection of CHKN2A staining. Surprisingly, the four tumors with significant CHKN2A upregulation in protein expression in tumor tissues also demonstrated no detection of CHKN2A staining in most of the deposited samples. These data could confirm the reported state of CDKN2A genetic alterations where these alterations manipulated the reflection of transcripts upregulation in tumor tissue when protein expression assessment and staining were considered.

One of the basic points for analysis in the current study and other CDKN2A-related reports was the analysis of CDKN2A genetic and epigenetic alterations under tumor status. CDKN2A loss was positively correlated with the aggressiveness of clinical behavior in mesotheliomas [50]. The state of CDKN2A was also employed for the diagnosis of follicular lymphoma as its loss was correlated with poor patient survival [51], and for soft tissue sarcoma where a poor outcome was also observed with CDKN2A deletion [28]. The deletion of CDKN2A was not only a diagnostic marker for a poor clinical outcome, but also it predicts metastasis of pancreatic tumor [52], recurrence of oral squamous cell carcinoma [53], and early recurrence in meningioma [54,55]. Matching with these findings, the current study found “deep deletion” as the most common form of the genetic alterations that occur to CDKN2A under tumor conditions where the assessment of alteration frequency in tumors, including GBM, MESO, SARC, and THYM, demonstrated “deep deletion” as almost the only form of investigated alterations. Additionally, “mutation” came in the second rank for CDKN2A alteration frequency assessment and represented almost one-third of reported alterations in cases with HNSC, PAAD, LUSC, and SKCM. Matching with our findings, CDKN2A mutations were predisposing to multiple nerve sheath tumors, melanoma, and internal malignancies [56]. Along with KRAS, TP53, SMAD4, BRCA1, and BRCA2, CDKN2A mutation was reported as the most frequent genetic alteration found in pancreatic cancer [57]. Additionally, TP53 and CDKN2A mutations were reported in never-smoker oral tongue squamous cell carcinoma and predicted poor clinical outcomes [58]. Moreover, cases with CDKN2A mutation had statistically significantly worse survival in comparison to melanoma cases with no CDKN2A mutations [59]. CDKN2A mutation was coupled with other genes mutation in different human cancers. For example, CDKN2A and BAP1 germline mutations predispose melanoma and mesothelioma [60], while CDKN2A and HRAS were frequently mutated in vulvar squamous cell carcinoma [61]. 

The second type of CDKN2A alteration, that was assessed in the current study, was epigenetic alteration. Hypermethylation has a role in carcinogenesis through the activation of the transcriptional silencing of tumor suppressor genes [62]. It was not a surprise that the assessment of CDKN2A CpG island methylation in our study exhibited hypermethylation in tumor tissue in comparison to the normal control in all of the analyzed human tumors with statistically significant results. Additionally, 16 human tumors, including BLCA, BRCA, CHOL, COAD, ESCA, HNSC, KIRC, LIHC, LUAD, LUSC, PAAD, PRAD, READ, SARC, UCEC, and CESC, demonstrated promoter hypermethylation of CDKN2A in tumor tissues in comparison to normal ones. Matching with our results, promoter hypermethylation was reported to inactivate CDKN2A in sporadic parathyroid adenomas [63], breast cancer [64], periocular sebaceous carcinoma [65], and hepatocellular carcinoma [66]. The state of CDKN2A hypermethylation and inactivation under tumor conditions predicts different adverse events. For example, Hypermethylated CDKN2A and CHFR were assessed as biomarkers for predicting radio resistance in esophageal squamous cell carcinoma [67]. Moreover, CDKN2A promoter hypermethylation was associated with increased cancer cell migration and tumor invasiveness in laryngeal squamous cell carcinoma [68], and cervical cancer [69]. In addition to that, CDKN2A promoter hypermethylation had a critical role in pancreatic carcinogenesis and can be employed as a prognostic marker [70].

Immune cells play major roles within the tumor micro-environment [71]. Additionally, the last few years had witnessed several advancements in the field of tumor immunotherapy [72]; therefore, it was important to investigate the correlation between CDKN2A alterations and the levels of several immune components. CD8 T cell plays a major role in controlling tumor progression [73], hence it was targeted with immune checkpoint inhibitors to reinvigorate this cytotoxic cell and fight against cancer [74]. Collectively, the infiltration and the exhaustion status of the CD8 T cell are important factors that control tumor growth [75], therefore the current study asked about the consequences of CDKN2A alterations under tumor conditions on the infiltration and the status of the CD8 T cell. Starting with the infiltration, none of the analyzed human tumors showed a positive correlation between CDKN2A deep deletion and the CD8 infiltration while on the other hand, HNSC, KICH, KIRC, LGG, LUAD, PAAD, SKCM, STAD, and THYM experienced a negative correlation between CDKN2A deep deletion and CD8 T cell infiltration. Moving to the CD8 exhaustion assessment, it is well established that the expression of the immune checkpoints is a basic marker of CD8 exhaustion [76,77,78]. In the current study, we assessed two sets of tumors for the exhaustion status of the infiltrated CD8, the first set included PAAD and HNSC which experienced a negative correlation between CDKN2A alterations and CD8 T cell infiltration, while the second set included BLCA and LUSC and did not experience the above-mentioned negative correlation. Importantly, the four tumors in the two sets demonstrated a positive correlation between the CDKN2A genetic alterations and the expression of five immune checkpoints namely PD-1, CTLA-4, LAG-3, TIM-3, and TIGIT. Hence, we can conclude that CDKN2A genetic alterations can generally direct tumor-infiltrated CD8 T cells to the exhaustion status where some of the analyzed human tumors experienced also a reduction in the CD8 infiltration while others did not experience that reduction. It is worth mentioning that the CD8 T cell was not the only affected immune component with the CDKN2A alteration in our study but also other chemokines and chemokine receptors, which modulate the state of tumor progression, were also affected. CCL4 is a chemokine that promotes cell proliferation, invasion, and migration of cancer [79]. Moreover, the enhancement of the CCL4-CCR5 axis was reported to promote glioblastoma invasion [80]. Additionally, high levels of CCL4 in the tumor microenvironment predict unfavorable survival in LUAD [43]. Matching with these cancer-promoting roles of the CCL4, the current study reports a positive correlation between CKN2A alteration and the expression of CCL4 in 10 tumors, namely BLCA, GBM, HNSC, LUAD, LUSC, MESO, PAAD, READ, SKCM, and STAD. CCR6 is a chemokine receptor that promotes tumor growth through the recruitment of tumor-promoting macrophages [81,82]. It can stimulate tumor angiogenesis via the AKT/NF-κB/VEGF pathway [83]. Its overexpression enables metastasis and poor clinical outcomes in different human cancers [84,85]. Matching with that, our study revealed a positive correlation between CDKN2A alterations and CCR6 in eleven analyzed human tumors. Collectively, CD8 T cell infiltration and exhaustion status, CCL4 and CCR6 expression are different immune components that were affected by the CDKN2A genetic alterations in a way that finally enables tumor growth and progression.

The final stage of the current study was the enrichment analysis of CDKN2A-related genes to investigate the molecular mechanism that allows the malfunction of CDKN2A, due to its genetic alterations, to direct the cell into carcinogenesis. The output of the enrichment analysis demonstrated that these genes are responsible for the regulation of the cell cycle and cell division. Moreover, two genes (E2FA and MCM2) were common ones in the two gene lists of CDKN2A-interacted and CDKN2A-correlated genes. E2Fs are important regulators of the cell cycle as they control the transcription of numerous genes functioning in DNA replication and cell cycle progression [86]. On the other hand, MCM2 regulates DNA replication, and its overexpression was detected in several human tumors where the dysfunctional MCM2 was correlated with tumor invasion and poor patient survival [87]. Collectively, CDKN2A interacts with several genes to regulate the cell cycle and division, and the epigenetic or genetic alterations to CDKN2A would have several impacts including unregulated cell division, manipulation of several immune components that finally stimulate tumor progression, and these factors lastly would enable a poor clinical outcome.

The last point to be discussed is the integration of the current study findings. We found that under several forms of human cancers, CDKN2A was highly expressed in cancerous tissue in comparison to healthy one and this feature was correlated with poor clinical outcome and positively correlated with the tumor stage, grade, and metastasis. A major concern that would appear is how the upregulation of a well-defined tumor suppressor gene could potentiate the tumor stage and grade and negatively affect the patient survival and to answer that question we analyzed the genetic alterations that could occur to CDKN2A under tumor conditions where we found that CDKN2A was highly mutated; therefore it lost its original function and acquired oncogene-like properties and the low intensity of CDKN2A staining in the cancerous human samples would confirm that as the highly expressed CDKN2A in tumor tissue is the mutated CDKN2A, not the original functioning one. Matching with that, we found promoter hypermethylation of CDKN2A in tumor tissue as an epigenetic control method to inhibit the expression of the “normal” functioning CDKN2A tumor suppressor gene in the tumor tissue. Therefore, we can conclude that under tumor conditions, CDKN2A faces epigenetic modifications to hinder its expression and only CDKN2A with loss-of-function mutations is highly expressed in the tumor tissue and that collectively results in a poor clinical outcome. This complex understanding of the CDKN2A status under tumor conditions is matched with previous reports that studied CD8 T cell memory formation after the removal of chronic viral infection [88,89] where researchers found that despite the generated memory cells have shown phenotypic and transcriptional characteristics very close to the formed memory cell under acute infection models, the former cells were epigenetically scared and still have features of the exhausted CD8 T cells which affected the recall capabilities of these cells. It is noteworthy that their studied model (chronic viral infection) and ours (cancer) is a chronic condition hence it seems that the human body deals with this chronic condition in a specific complex manner. 

## 5. Conclusions

CDKN2A is categorized as a major tumor suppressor gene where the genetic and epigenetic alterations that usually occur to that gene can be correlated with poor patient survival in different types of human tumors. These alterations modulate several immune components, including the infiltration and the exhaustion status of the CD8 T cells in addition to the expression of the chemokine CCL4 and the chemokine receptor CCR6 to finally potentiate the tumor growth. These alterations also inhibit the cell cycle regulation roles of CDKN2A which allows for tumor initiation. Therefore, the genetic and epigenetic alterations of CDKN2A can be employed as an immunological and prognostic biomarker in a pan-cancer model.

## Data Availability

The data presented in this study are available on request from the corresponding author.
